# Nonsteroidal anti-inflammatory drugs modulate cellular glycosaminoglycan synthesis by affecting EGFR and PI3K signaling pathways

**DOI:** 10.1038/srep43154

**Published:** 2017-02-27

**Authors:** Paweł Mozolewski, Marta Moskot, Joanna Jakóbkiewicz-Banecka, Grzegorz Węgrzyn, Katarzyna Bocheńska, Bogdan Banecki, Magdalena Gabig-Cimińska

**Affiliations:** 1 Department of Molecular Biology and Genetics, University of Gdańsk, Wita Stwosza 59, 80-308 Gdańsk, Poland; 2 Institute of Biochemistry and Biophysics, Polish Academy of Sciences, Laboratory of Molecular Biology (affiliated with the University of Gdańsk), Wita Stwosza 59, 80-308 Gdańsk, Poland; 3 Department of Molecular Biology, University of Gdańsk, Wita Stwosza 59, 80-308 Gdańsk, Poland; 4 Department of Molecular and Cellular Biology, Intercollegiate Faculty of Biotechnology UG-MUG, Abrahama 58, 80-307 Gdańsk, Poland

## Abstract

In this report, selected non-steroidal anti-inflammatory drugs (NSAIDs), indomethacin and nimesulide, and analgesics acetaminophen, alone, as well as in combination with isoflavone genistein as potential glycosaminoglycan (GAG) metabolism modulators were considered for the treatment of mucopolysaccharidoses (MPSs) with neurological symptoms due to the effective blood-brain barrier (BBB) penetration properties of these compounds. We found that indomethacin and nimesulide, but not acetaminophen, inhibited GAG synthesis in fibroblasts significantly, while the most pronounced impairment of glycosaminoglycan production was observed after exposure to the mixture of nimesulide and genistein. Phosphorylation of the EGF receptor (EGFR) was inhibited even more effective in the presence of indomethacin and nimesulide than in the presence of genistein. When examined the activity of phosphatidylinositol-3-kinase (PI3K) production, we observed its most significant decrease in the case of fibroblast exposition to nimesulide, and afterwards to indomethacin and genistein mix, rather than indomethacin used alone. Some effects on expression of individual GAG metabolism-related and lysosomal function genes, and significant activity modulation of a number of genes involved in intracellular signal transduction pathways and metabolism of DNA and proteins were detected. This study documents that NSAIDs, and their mixtures with genistein modulate cellular glycosaminoglycan synthesis by affecting EGFR and PI3K signaling pathways.

Glycosaminoglycans (GAGs, formerly called mucopolysaccharides), endogenous organic compounds synthesized in most tissues of human body, play important roles in fundamental biological processes, mostly by binding to variety of proteins^1^. In normal cells there is a constant turnover of GAGs in lysosomes, which is mediated by specific acid hydrolases. However altered GAG metabolism is responsible for multiple cell, tissue and organ damages throughout the body. Currently identified diseases of this type include mucopolysaccharidoses (MPSs), a group of genetically determined metabolic disorders. Depending on specific enzymatic defects, in various types of MPS, lysosomal storage of particular GAG or their combination occurs. The growing understanding of the etiology of these diseases has led to the development of specific therapeutic approaches. The methods rely on direct (enzyme replacement therapy - ERT) or indirect (hematopoietic stem cells’ transplantation - HSCT, gene therapy - GT) delivery of the appropriate lysosomal enzyme into the patient. Research has shown that supplementing the absence or deficiency of active enzyme directly or indirectly via the above-mentioned treatment methods can indeed cause degradation of accumulated glycosaminoglycans, which in turn translates into improved clinical status of a patient^2^. However, these approaches have their specific limitations^3^. For example, the major problem of ERT concerns the inefficient enzyme dissemination to all occupied sites in the body, including its ineffective delivery to the central nervous system (CNS) due to an ineffective delivery of proteins through the blood-brain barrier^2^. Another group of treatment includes a method based on reduced synthesis of substrate (called substrate reduction therapy - SRT)^4^. This strategy relates to the partial inhibition of enzymes of the lysosomal substrate biosynthetic pathway by low molecular weight inhibitor, leading to reduction of these *de novo* synthesized substrates. The purpose of this is to restore in pathologically changed cells the equilibrium between the processes of synthesis and degradation of these substrates. This type of approach tries to apply in various cases of lysosomal storage diseases, including the neurodegenerative disorders^4^.

Recently, studies on the implementation of the concept of substrate reduction therapy in the treatment of MPS were conducted^5^,^6^. The work has been performed on the use of various compounds to lower yield of glycosaminoglycans and being effective in treatment of neuronopathic forms of MPSs due to their ability to cross the BBB. So far several flavonoids were tested, and among them, genistein has been studied most intensively, and it was proposed that this compound can downregulate GAG production by blocking phosphorylation of the epidermal growth factor (EGF) receptor (EGFR), thus impairing a signal transduction pathway necessary for activation of genes coding for enzymes involved in this anabolic process^7^,^8^,^9^. Indeed, we identified the genistein-mediated gene network regulating not only GAGs biosynthesis, but also degradation of these macromolecules, taking into consideration the entire lysosomal metabolism^10^,^11^,^12^. This work led us to revise the action of the other EGFR inhibitors, which could be considered as putative drugs in MPS treatment, even more efficient in treating the neurological symptoms. This will be a milestone in the development of substrate reduction therapy and crucial in view of fact that the SRT is a promising strategy for treatment of mucopolysaccharidoses, and what is most important, it gives chance to be effective for their neurodegenerative forms, for which no treatment is available at the moment. The agents being candidates should be relatively weak inhibitors, while GAGs are compounds necessary for development and proper function of many tissues and organs, thus, complete or very strong inhibition of their products would not be desired, especially in children treatment. Additionally, SRT for inherited metabolic diseases, like MPS, must be considered as a long-term therapy, potentially for the whole life, thus agents with at most marginal adverse effects are considered. The investigations showed that acetaminophen (*N*-(4-hydroksyfenylo)acetamid), indomethacin (2-{1-[(4-chlorophenyl)carbonyl]-5-methoxy-2-methyl-1H-indol-3-yl}acetic acid) and nimesulide *N*-(4-Nitro-2-phenoxyphenyl)methanesulfonamide) are substances remarkably inhibiting glycosaminoglycans synthesis^13^,^14^. While the first one is analgesics, the two others belong to nonsteroidal anti-inflammatory drugs (NSAIDs), commonly used for the reduction of pain, fever, inflammation and stiffness. This information, together with their analgesic and anti-inflammatory action speaks for their potential use in treatment of diseases, such as mucopolysaccharidoses, usually accompanied by strong pain and inflammation. Therefore, in this study, the above mentioned compounds, as well as their mixtures with genistein (previously investigated in the context of lysosomal modulation^10^,^11^), were selected and applied for investigation of their effects on GAG metabolism in cultures of healthy and MPS cells. The implementation of a cell culture model, that shows a finite proliferative capacity has the advantage of providing a controlled environment to investigate a wide variety of cellular phenomena. It has also the inherent limitation of isolating cells from the regulatory elements that might be provided by other types of cells. Among different cells types (such as keratinocytes, melanocytes, lymphocytes, glial, lens and endothelial cells), fibroblasts from normal and MPS individuals exhibit most limited replicative life span in the culture, and therefore, remain a powerful tool for our studies.

## Materials and Methods

### Study approval

All methods were carried out in accordance with guidelines and regulations included in the official documents of the University of Gdańsk. All experimental protocols were approved by the Research Committee of the Department of Molecular Biology of the University of Gdańsk.

Skin fibroblasts were obtained from humans who had given informed consent at the Children’s Memorial Health Institute, Warsaw, Poland.

### Cell lines, culture media and reagents

Human Dermal Fibroblasts, adult (HDFa) (Cascade Biologics, Portland, USA) and MPS fibroblasts types IIIA, IIIB and VI (Children’s Memorial Health Institute, Warsaw, Poland) were cultured in Dulbecco’s modified Eagle’s medium (Thermo Fisher Scientific Inc., Paisley, UK) supplemented with 10% fetal bovine serum (FBS) and 1% antibiotic/antimycotic solution (Sigma-Aldrich Co. LLC., St. Louis, USA) at 37 °C in a humidified atmosphere containing 5% carbon dioxide (CO_2_). Jurkat T cells (American Type Culture Collection, Manassas, USA) were grown in RPMI-1640 medium (Thermo Fisher Scientific Inc., Paisley, UK) supplemented with 10% FBS and 1% antibiotic/antimycotic solution (Sigma-Aldrich Co. LLC., St. Louis, USA) at 37 °C in a humidified atmosphere containing 5% CO_2_. Viability of Jurkat T cells was assessed using MUSE^®^ Cell Analyzer (Merck Millipore, Germany) using a Count & Viability Assay Kit (Merck Millipore, Germany) before subculturing or experimental procedure. Wortmannin (Sigma-Aldrich Co. LLC., St. Louis, USA) prepared in dimethyl sulfoxide (DMSO, Sigma-Aldrich Co. LLC., St. Louis, USA) and stored at a stock concentration of 2 mM at −20 °C was used for inhibition of constitutively active PI3K signaling pathway in Jurkat T cells. Used in this study NSAIDs, i.e. acetaminophen, indomethacin, nimesulide (Sigma-Aldrich Co. LLC., St. Louis, USA), and isoflavone genistein (Pharmaceutical Research Institute Warsaw, Poland) were dissolved in DMSO, and added in the indicated final concentrations, in the case of NSAIDs as stated in literature and reports on the clinical usage^13^,^15^,^16^,^17^,^18^, while for genistein as determined in our previous studies on cell cultures^8^.

### Cytotoxicity and proliferation assays

Cell viability was assessed with MTT assay. For this, 6 × 10^3^ cells per well (in cytotoxicity assay) or 10^3^ cells per well (in proliferation assay) were seeded in flat-bottomed 96-well plates and grown overnight at 37 °C and 5% CO_2_. Then the growth medium was substituted with fresh one supplemented with tested compounds at appropriate concentrations or 0.05% DMSO as control (Ctrl). After period of time (i.e. 24 and 48 h to test cytotoxicity, or for 7 days to test cell proliferation), medium was replaced with RPMI-1640 (Sigma-Aldrich Co. LLC., St. Louis, USA) supplemented with MTT (3-[4,5-dimethylthiazol-2-yl]-2,5-diphenyltetrazolium bromide, 1 mg/ml) (Sigma-Aldrich Co. LLC., St. Louis, USA) for another 4 h. The purple formazan crystals were dissolved in 150 ml of 100% DMSO, and absorbance was determined at 570 nm using Wallac 1420 Multilabel Counter (Perkin Elmer Inc., MA, USA).

### Measurement of kinetics of GAG synthesis

Glycosaminoglycan synthesis was estimated by measurement of ^35^S or ^3^H incorporation into GAG chains. Cells were plated in a number of 2 × 10^4^ per well in 48-well plate and incubated overnight to allow the attachment. Next, cells were treated for 48 hours in standard DMEM supplemented with appropriate amounts of particular NSAIDs, genistein, mixtures of selected NSAIDs with genistein or DMSO only (Ctrl). After 48 h of incubation, cells were labelled for 24 hours with 20 μCi/ml of H_2_[^35^S]O_4_ or 10 μCi/ml of ^3^H-GlcN (Hartmann Analytic GmbH, Braunschweig, Germany) in growth medium without inorganic sulfates (Minimum Essential Medium Eagle, Joklik’s modified, Sigma-Aldrich Co. LLC., St. Louis, USA) or growth medium without glucose (Thermo Fisher Scientific Inc., Paisley, UK) mixed with standard DMEM medium (1:1) supplemented with tested compounds or DMSO. Next, cells were washed six times with Dulbecco’s phosphate-buffered saline (DPBS, Thermo Fisher Scientific Inc., Paisley, UK) and digested for 3 hours at 65 °C with 0.5% papain (Sigma-Aldrich Co. LLC., St. Louis, USA) (prepared in 200 mM phosphate buffer [Na_2_HPO_4_ - NaH_2_PO_4_], pH 6.4, containing 100 mM sodium acetate, 10 mM Na_2_EDTA and 5 mM L-cysteine). Incorporation of radioactive precursors was measured in a MicroBeta^2^ scintillation counter (Perkin Elmer Inc., MA, USA) and Quant-iT™ PicoGreen^®^ dsDNA Reagent (Thermo Fisher Scientific Inc., Paisley, UK) was used to determine DNA concentration in papain digested samples. Incorporation of radioactive precursors was calculated per DNA amount (cpm/ng of DNA) and expressed as relative to control cultures (treated with 0.05% DMSO only).

To study the effects of a combination of tested compounds in order to provide evidence of a significant superiority compared to the single agents the Combination Index (CI) with the use of the Bliss Independence model was determined^19^,^20^. It is based on the principle that drug effects are outcomes of probabilistic processes and assumes that drugs act independently in such a manner that neither of them interferes with the other (different sites of action), but each contributes to a common result. CI is recognized as the standard measure of combination effect that indicated a greater (CI < 1), lesser (CI > 1) or similar (CI = 1) effect than the expected additive effect.

### EGFR and PI3K phosphorylation analysis

Efficiency of phosphorylation inhibition of the epidermal growth factor receptor (EGFR) or phosphoinositide 3-kinase (PI3K) pathway was assessed using MUSE^®^ Cell Analyzer (Merck Millipore, Germany) using a Millipore’s Muse™ Activation Dual Detection kits (Merck Millipore, Germany). HDFa cells were seeded in 6-well plates at a density of 2 × 10^5^ per well. Following an overnight incubation, standard growth medium was substituted with the medium containing 0.05% DMSO only, supplemented with 100 ng/ml EGF in 0.05% DMSO, or with 100 ng/ml EGF in 0.05% DMSO and particular tested compounds at the indicated final concentrations^21^. After 24 hours EGFR pathway activation was determined by Muse^®^ EGFR-RTK Activation Dual Detection kit (Merck Millipore, Germany) by using a phospho-specific anti-phospho-EGFR (Tyr1173)-Alexa Fluor^®^555 and an anti-EGFR-PECy5 according to the manufacturer’s protocol. Similarly, PI3 kinase pathway activation was assessed using an anti-phospho-Akt (Ser473), Alexa Fluor^®^555 and an anti-Akt, PECy5 provided in the PI3K Activation Dual Detection kit (Merck Millipore, Germany). Additionally, Jurkat T cells treated with 0.5 μM wortmannin were used as a control of PI3K activation status determination.

### Total RNA extraction

For transcriptomic analyses 1 × 10^5^ cells were seeded and grown for 24 h. Then, the medium was substituted with either NSAIDs-free one, containing DMSO (Ctrl), or the one supplemented with appropriate amounts of tested drug, genistein or mixture of drug and genistein. Total RNA was extracted from cells using the High Pure RNA Isolation Kit (Roche Applied Science, IN, USA) and quantified with the Quant-it^TM^ RiboGreen^®^ assay kit (Thermo Fisher Scientific Inc., Paisley, UK) following the manufacturer’s instructions. In addition, the quality of each RNA sample was assessed using the RNA 6000 Nano Assay on the Agilent 2100 Bioanalyzer (Agilent Technologies Inc., CA, USA).

### DNA microarray processing and real-time quantitative RT-PCR assays for mRNA analysis

Whole genome microarray analysis of three biological replicates was performed using Illumina’s Human HT-12v4 Expression BeadChips, targeting more than 25,000 genes with more than 48,000 probes (Illumina Inc., CA, USA), for all tested conditions. BeadChips were scanned using an Illumina BeadArray Reader and the Bead Scan Software (Illumina Inc., CA, USA). The quality of microarray data was controlled by examining raw and adjusted intensity histograms. The assay performance and data extraction was done according to Moskot *et al*.^10^ All gene expression data have been deposited in the NCBI Gene Expression Omnibus (GEO series accession number GSE63239), according to the MIAME (minimum information about a microarray experiment) standards. An overview of experiment performance was gained by clustering samples using a correlation metric (Illumina^®^ BeadStudio Data Analysis software). The Pearson correlation coefficient method was used to calculate “expression distance values” across experiments and to group samples that have similar expression patterns. The values ranging between 0.98 and 0.99 for biological replicates indicate a high degree of reproducibility and strong correspondences between expression profiles.

Quantitative real-time Reverse Transcription PCR (real-time qRT-PCR) was performed to measure the mRNA levels of the studied genes. Total RNA was reverse-transcribed into cDNA using Transcriptor First Strand cDNA Synthesis Kit (Roche Applied Science, IN, USA), according to the manufacturer’s instructions. Real-time qRT-PCR was carried out with Real-Time ready Custom Panel (cat. no. 05532914001, config. no. 100093799; Roche Applied Science, IN, USA) and the LightCycler^®^ 480 Probes Master (Roche Applied Science, IN, USA) using the Light Cycler 480 II detection system (Roche Applied Science, IN, USA). Expression values were normalized against three control genes *ACTB, SDHA* and *YWHAZ* of constant expression level (2^−ΔΔct^ method). Determination of reference genes for real-time qRT-PCR was assessed using commercially available RealTime ready Human Reference Gene Panel (cat no. 05339545001, Roche Applied Science, IN, USA). Moreover, statistical analyses of the normalized gene expression data were performed in Prism (GraphPad). For both DNA microarray and real-time qRT-PCR analyses, a fold change (FC) greater or equal to 1.3 and below and equal 0.7 was considered as a relevant criterion for genes being significantly differentially expressed, with a *p* value of <0.05.

### Gene Ontology and Gene Set Enrichment studies

The transcriptomic analysis was conducted through combining gene set enrichment analysis (GSEA)^22^, with the use of GOrilla^23^ and REViGO (visual interaction gene network analysis)^24^ tools for reconstructing crucial molecular networks and target genes identification. For GSEA study goals Reactome was used as reference database for ontology distribution with FDR *q*-value below 0.1 overall as a limitation for ontology selection. Tests for enrichment of genes with shared Biological Process Gene Ontology (GO) terms were performed using the online tool GOrilla. REViGO was used to eliminate redundant GO terms, and multiple-test correction for significant GO. Genes exhibiting fold change ≥1.3 and below and equal 0.7 with a *p* value less than 0.05, defined as differential expressed genes (DEGs), were examined.

## Results

### Selection of analgesic and nonsteroidal anti-inflammatory drugs and study of their cytotoxic and antiproliferative activity

It has been previously demonstrated that acetaminophen, indomethacin and nimesulide are among analgesics and NSAIDs which inhibit GAG synthesis^13^,^14^,^25^. Moreover, mixtures of those compounds with genistein, flavonoid responsible for impairment of production and enhancement of degradation of glycosaminoglycans^6^,^10^,^11^, were included in this work in order to see whether they might result in even more effective reduction of GAG accumulation than the use of any of these compounds alone. The concentrations of acetaminophen (5–500 μM), indomethacin (1.5–50 μM), and nimesulide (0.3–100 μM) used are close to clinically relevant molar concentrations, while genistein of 100 μM as previously applied^6^,^10^,^11^.

**Figure 1:**
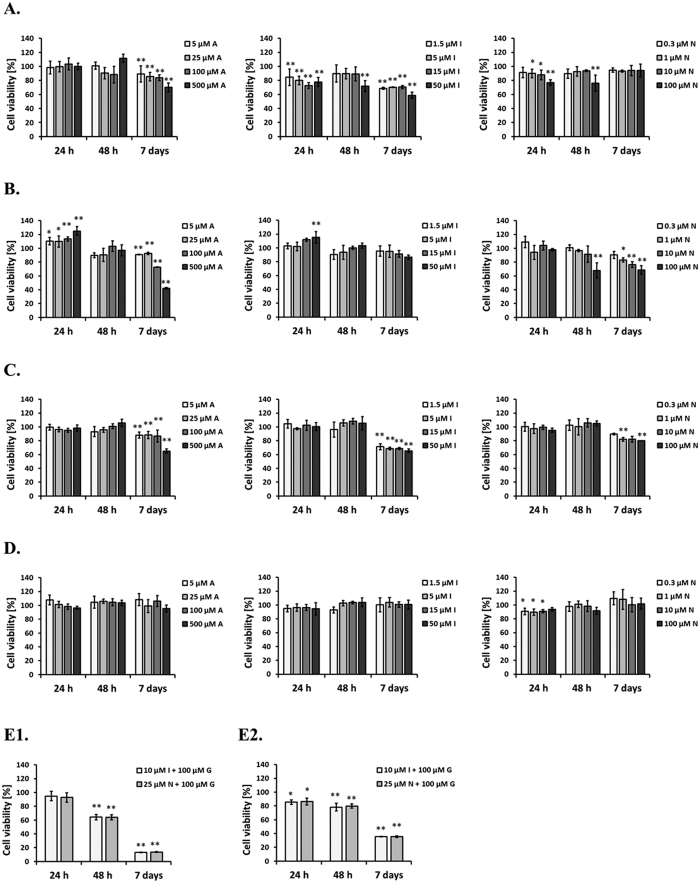
Cytotoxic and antiproliferative activity of acetaminophen (**A**), indomethacin (I), and nimesulide (N), respectively, in HDFa (panel A), MPS IIIA (panel B) and IIIB (panel C), and MPS VI (**D**) fibroblasts. Sections E1 and E2 stand for viability of HDFa and MPS IIIA cells treated with mixtures of indomethacin (I) and genistein (G), and also nimesulide (N) and genistein (G). Statistically significant differences in cell viability relative to control cells (treated with 0.05% DMSO only) are indicated with * for *p* < 0.05, ** for *p* < 0.01. Statistical analysis was performed using ANOVA with Tukey’s HSD Post Hoc test.

We first tested selected drugs in order to establish their cytotoxicity and antiproliferative activity in cultured fibroblasts. For this, we measured metabolic activity of HDFa, MPS IIIA, IIIB and VI cells treated with different concentrations of acetaminophen (5, 25, 100, and 500 μM), indomethacin (1.5, 5, 15, and 50 μM), or nimesulide (0.3, 2, 10, and 100 μM) for different periods of time (24, 48 h, and 7 days). This study demonstrated that 24 and 48 h treatment with clinically achievable low concentrations of tested compounds has little cytotoxic effect (Fig. 1). Additionally, no remarkable inhibitory effect of particular compounds on fibroblasts growth after 7 days was visible, while clear antiproliferative activity was observed for mixtures used, i.e. both indomethacin or nimesulide with genistein (Fig. 1).

### Assessment of the effectiveness of inhibition of GAG synthesis by acetaminophen, indomethacin, nimesulide, and their mixtures with genistein

**Figure 2:**
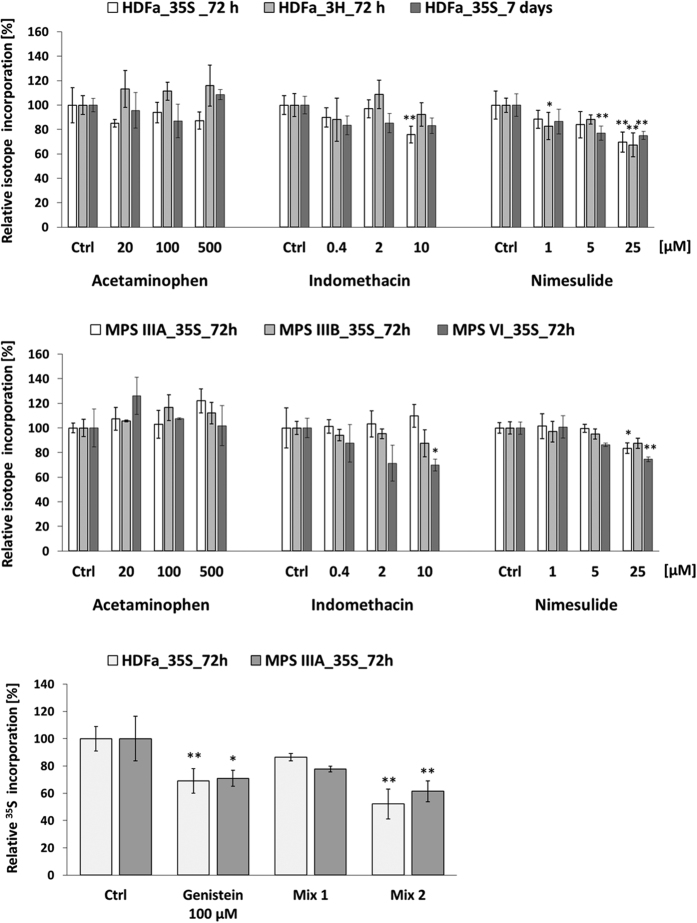
Relative level of glycosaminoglycan synthesis in HDFa, MPS IIIA, IIIB and VI fibroblasts after 72 h or 7-day treatment with different concentrations of acetaminophen, indomethacin, nimesulide, genistein (100 μM), mix 1 (10 μM indomethacin + 100 μM genistein), and mix 2 (25 μM nimesulide + 100 μM genistein), measured by incorporation of [^35^S]O_4_^2−^ or [^3^H]GlcN. Radioactivity of incorporated precursors was quantified in a scintillation counter, calculated per DNA amount [dpm/ng DNA], and expressed as the percentage of control (‘Ctrl’ stands for cell culture treated with 0.05% DMSO). Statistical analysis was performed by using ANOVA with Tukey’s HSD Post Hoc test. Significance values are indicated with * for *p* < 0.05, ** for *p* < 0.01.

The level of GAG synthesis was estimated by measurement of [^35^S]O_4_^2−^ or [^3^H]GlcN uptake in both HDFa and various types of MPS fibroblasts. Tested compounds were added individually at the final concentration of 20, 100, and 500 μM for acetaminophen, 0.4, 2, and 10 μM for indomethacin and 1, 5, and 25 μM for nimesulide, 100 μM for genistein, or applied as mix of 10 μM indomethacin and 100 μM genistein, or 25 μM nimesulide and 100 μM genistein to cell culture. We found that two of all tested compounds, i.e. indomethacin and nimesulide, but not acetaminophen, revealed ability to inhibit significantly GAG synthesis in HDFa, MPS IIIA and VI (Fig. 2). The most pronounced impairment of glycosaminoglycan production measured by estimation of the amount of incorporated ^35^S was observed after 72-hour exposure to nimesulide and genistein mixture in HDFa (48% decrease) and MPS IIIA (40% reduction). Furthermore, statistically significant differences in GAG synthesis relative to untreated cells were documented by estimation of the amount of incorporated ^35^S with some exceptions as follows: (i) at the level of around 30% reduction for HDFa treated for 72 hours with 25 μM nimesulide (when both radioactive precursors applied), HDFa and MPS IIIA treated for 72 hours with 100 μM genistein, and MPS VI in the presence of 10 μM indomethacin; (ii) at the level of 25% decrease for HDFa treated for 72 hours with 10 μM indomethacin and for 7 days with 25 μM nimesulide, as well as for MPS VI exposed to 25 μM nimesulide for 72 hours; and (iii) at the level of 15–20% decline for HDFa treated for 72 hours with 1 μM nimesulide (glucosamine, D-[1-3 H] incorporation), and for 7 days with 5 μM nimesulide, as well as for MPS IIIA exposed to 25 μM nimesulide for 72 h. Interestingly, acetaminophen stimulated rather than inhibited GAG synthesis (Fig. 2). In addition, the Combination Index (CI) with the use of the Bliss Independence model was determined for the action of tested mixtures in HDFa and MPS IIIA fibroblasts. For both cell lines the CI value was 1.9 in the case of indomethacin and genistein combination indicating an antagonism. When the mixture of nimesulide and genistein was used, the CI was equal to 1.0, resulting in an additive effect.

### Inhibition of phosphorylation of EGF receptor in the presence of indomethacin, nimesulide and genistein

**Figure 3:**
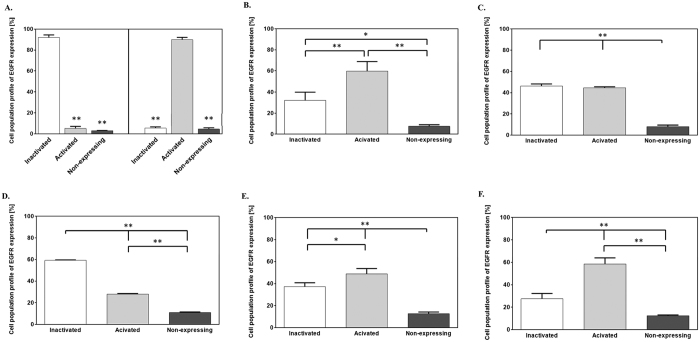
Effects of investigated NSAIDs and their mixtures with genistein on tyrosine kinase activity of EGF receptor (EGFR). HDFa cells were treated with 0.05% DMSO and used as control (A, left panel), with 100 ng/ml EGF in 0.05% DMSO (A, right panel), or with 100 ng/ml EGF in 0.05% DMSO and particular compounds alone such as 100 μM genistein (**B**), 10 μM indomethacin (**C**), 25 μM nimesulide (**D**), or in mixtures of 10 μM indomethacin and 100 μM genistein (**E**), or 25 μM nimesulide and 100 μM genistein (**F**). Percentage of inactivated cells, activated cells (via EGFR phosphorylation) and non-expressing cells was determined for each experimental condition. Data represent the average and standard deviation of three independent experiments. Statistical analysis was performed by using one-way ANOVA and the Tukey’s HSD Post Hoc test. Values of *p* < 0.05 (*) or *p* < 0.01 (**) are indicated.

To test whether compounds used in this study impair GAG synthesis by the same mechanism as that proposed for genistein, we measured efficiency of phosphorylation of EGFR in the presence and absence of investigated NSAIDs, i.e. indomethacin and nimesulide, effectively inhibiting GAG synthesis in fibroblasts. In addition, tests with the use of mixture of indomethacin and genistein, and also nimesulide and genistein were performed. The extent of EGFR pathway activation by measuring EGFR phosphorylation relative to the total EGFR expression in given cell population was investigated. By doing such, the levels of both the total and phosphorylated protein were measured simultaneously in the same cell, resulting in a normalized and accurate measurement of EGFR activation after drug stimulation. We found that both NSAIDs tested separately revealed statistically significant inhibition of EGFR phosphorylation, even more effective than that of genistein, or compounds’ combinations (Fig. 3).

### PI3K activity decrease in fibroblasts treated with NSAIDs and genistein

**Figure 4:**
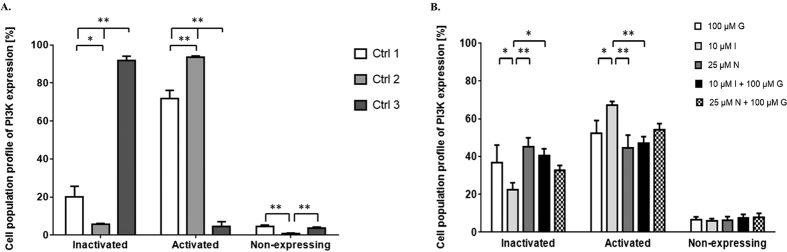
Activity of phosphatidylinositol-3-kinase (PI3K) in HDFa treated with 0.05% DMSO only (Ctrl 1), 10 μM indomethacin, 25 μM nimesulide, 100 μM genistein, or mixture of 10 μM indomethacin and 100 μM genistein, or 25 μM nimesulide and 100 μM genistein. Two additional control assays were included, Jurkat cells untreated (Ctrl 2) or treated with wortmannin (Ctrl 3). Percentage of inactivated cells, activated cells (via PI3K phosphorylation) and non-expressing cells was determined for each experimental condition. The results presented are average values obtained from three different experiments with bars indicating standard deviation. Statistical analysis was performed by using one-way ANOVA and the Tukey’s HSD Post Hoc test. Statistically significant differences in cell viability relative to control cells (treated with 0.05% DMSO only) are indicated with * for *p* < 0.05, ** for *p* < 0.01.

In this part of the work we studied further how the inhibition of EGFR phosphorylation by investigated NSAIDs influences the activity of phosphatidylinositol-3-kinase, a downstream effector of EGF receptor, in fibroblasts treated with particular indomethacin and nimesulide alone or with their mixture with genistein. When examined the activity of PI3K produced we observed its most significant decrease in the case of cell exhibition to nimesulide. Indomethacin in combination with genistein resulted in more effective inhibition of PI3K activity than used alone, however still less pronounced when compared to nimesulide (Fig. 4).

### Activity of genes involved in GAG and lysosomal metabolism, prostaglandin synthesis and intracellular signaling pathways

Microarray analyses were performed on HDFa cells after 24 and 48 h of either vehicle or particular drug treatment, i.e. with 2 and 10 μM indomethacin, or 5 and 25 μM nimesulide. In general, the microarray studies revealed that the fibroblasts responded to this treatment rather with minor changes in global expression profiles of only few dozen of genes, slightly modulating transcript level of genes involved in GAG metabolism pathway and lysosomal function, affecting however a number of genes related to different signal transduction pathways and metabolism of DNA and proteins, substantially when high concentrations of the NSAIDs were applied.

We used a real-time quantitative RT-PCR approach to examine in more detail the expression patterns of genes involved in GAG metabolism, as well as in lysosome biogenesis and function, whose activities were modulated in fibroblasts at tested conditions, as observed in microarray studies. Because significant changes in the activity of these genes were generally visible to the cells treated with 25 μM nimesulide, 100 μM genistein, and the mix of these two compounds, we implemented such conditions for real-time qRT-PCR analyses. Among 45 genes covered by Real Time ready Custom Panel, 7 of GAG metabolism pathways (5 of GAG synthesis and 2 of GAG degradation) and 7 sequences coding for lysosomal proteins (except for these 2 of GAG degradation), all revealing modulated activity in transcriptomic analyses, were detected (Table 1). Interestingly, we found an increase in mRNA level of *TFEB* (gene coding for transcription factor EB; 1.3 ± 0.1 v. *ACTB* and 1.7 ± 0.2 v. *YWHAZ* for mix of 10 μM indomethacin and 100 μM genistein, 1.7 ± 0.1 v. *ACTB* and 1.6 ± 0.0 v. *YWHAZ* for mix of 25 μM nimesulide and 100 μM genistein), and *MTOR* (gene coding for Serine/Threonine-Protein Kinase MTOR; 1.3 ± 0.2 v. *ACTB* and 1.6 ± 0.2 v. *YWHAZ* for mix of 10 μM indomethacin and 100 μM genistein, 1.5 ± 0.1 v. *ACTB* and 1.4 ± 0.1 v. *YWHAZ* for mix of 25 μM nimesulide and 100 μM genistein), and no changes in *EGFR* transcript amount compared with the untreated samples, in HDFa cells treated for 24 h with both mixtures, 10 μM indomethacin with 100 μM genistein, and 25 μM nimesulide with 100 μM genistein, in respect to *ACTB* and *YWHAZ* references, correspondingly (data not shown). At the same time, we did not notice any significant variations in the activity of all those three genes in cells treated individually with indomethacin or nimesulide (data not shown).

Moreover, no transcriptomic data regarding modulation of expression of prostaglandin synthesis were available due to the problem with low level analysis being related to elimination of the results for these transcripts after background adjustment. This could have resulted from the use of uninflamed, wild type HDFa fibroblasts, i.e. not induced by inflammatory stimuli. Though, we used a real-time quantitative RT-PCR approach to examine in more detail the expression patterns of two prostaglandin synthase genes, *PTGS1* and *PTGS2*, in fibroblasts exposed not only to 10 μM indomethacin or 25 μM nimesulide as it was in the case of microarray analysis, but also to their mixtures with 100 μM genistein. We found that indomethacin, but not nimesulide induced a 1.3-fold decrease of PTGS1 mRNA level, while *PTGS2* expression was down-regulated by nimesulide, not however by indomethacin, when compared with the non-treated counterpart by using both the *ACTB* and *YWHAZ* reference control genes. Peculiarly, the analysis showed that both genes *PTGS1* and *PTGS2* were however significantly enriched in their transcript level in response to the compound combinations’ treatment of cells.

**Figure 5:**
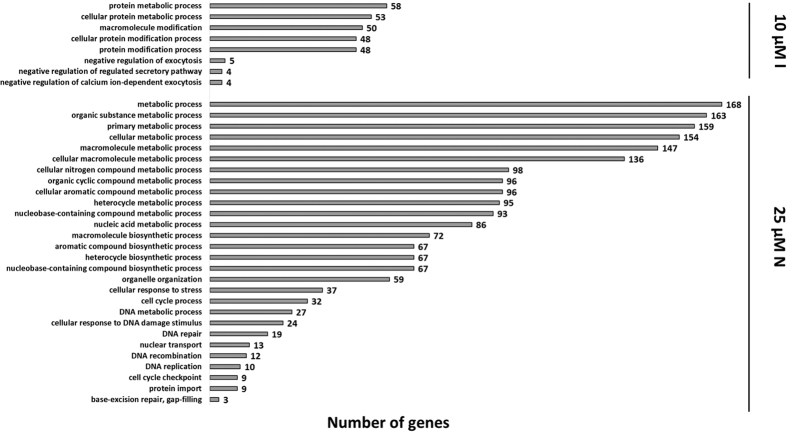
Significantly overrepresented GO terms, reflecting biological processes upon 10 μM indomethacin (10 μM I) or 25 μM nimesulide (25 μM N) treatment for 24 hours of HDFa cells, with false discovery rate (FDR) <0.1, fold change ≥1.3 and below and equal 0.7, and *p* < 0.001.

In addition, our studies revealed that selected NSAIDs and/or their combination with genistein can significantly modulate mRNA level of genes coding for products involved in regulation of cellular signaling pathways, and DNA and protein metabolism (Fig. 5 and Table 2). Gene Ontology analyses performed with the GOrilla tool of microarray data revealed that at the top of the list of NSAID-regulated genes, were those mainly involved in metabolic processes (for both 10 μM indomethacin and 25 μM nimesulide 24 h treatment) in the cell (Fig. 5). Accordingly, GSEA conducted on the Reactome pathways showed a significant enrichment of signal transduction cascades genes (involved mainly in the Wnt, TCR, SCF/c-kit associated with PI3K signaling), and among DNA and protein metabolism genes, regulated by 10 μM indomethacin, and also 25 μM nimesulide, after 24 h exposition (Table 2).

## Discussion

This work exploits and expands on recent breakthroughs in the understanding of cellular cross-talk to develop novel therapeutic approaches based on the use of analgesic nonsteroidal anti-inflammatory drugs to treat rare inherited lysosomal diseases, such as mucopolysaccharidoses caused by altered glycosaminoglycan metabolism. The encouraging results of research on the molecular mechanism of isoflavone genistein-mediated impairment of GAG production prompted us to continue the study on the implementation of the non-enzymatic SRT using GAG synthesis inhibitors to treat MPS, especially those with neurological symptoms^7^,^10^,^11^. Furthermore, the latest investigations exhibited that among different analgesics and NSAID compounds acetaminophen, indomethacin and nimesulide are substances remarkably inhibiting GAG synthesis^13^,^14^. It has been shown that the reason for this glycosaminoglycan level reduction is a deficiency of cellular sulphates, a key factor balancing the production and degradation of GAGs, resulting from NSAID medication with side-effect in form of sulphate depletion, which in turn takes place in the treatment of arthritis^25^,^26^,^27^. There is different matter in the case of patients suffering from mucopolysaccharidosis. The excess of GAGs, due to lack of or significantly reduced activity of one of the enzymes responsible for degradation of these compounds, affects cells, tissues, organs, and consequently the whole body. Thus, the performance of GAG synthesis reduction through the use of agents causing the sulphate depletion in the human body may yield in a related effect to that observed in the case of genistein. It is reported in the literature that the main mechanism of action of indomethacin and nimesulide is the inhibition of prostaglandin synthesis by blocking the activity of the enzyme cyclooxygenase (COX) isoforms, officially known as prostaglandin-endoperoxide synthase (PTGS) isoforms, COX-1 (PTGS1; non-selectively blocked by indomethacin), COX-2 (PTGS2; non-selectively blocked by indomethacin and selectively blocked by nimesulide) and/or COX-3 (selectively blocked by acetaminophen), which in turn leads to reduced signs of inflammation^28^. Another work, describing stimulation of angiogenesis, cell proliferation and differentiation in carcinogenesis by prostaglandins, indicated their additional function as activators of intracellular signaling pathway through EGF receptor^26^. Based on this, a hypothesis can be considered that the reduction of prostaglandin level, resulting from the inhibition of their synthesis in the cell exposed to the above substances responsible for the COX activity decrease, might lead to the inhibition of EGF receptor activity, thereby blocking the cellular signal transduction process essential for the expression of genes encoding the relevant enzymes involved in the metabolism of GAGs. However, this phenomenon has remained unclear. Therefore, we intended to validate the extent of action of not only flavonoids, but also selected drugs, and to learn interactions at the molecular level in terms of modulation of cellular responses by these substances.

As acetaminophen, indomethacin and nimesulide belong to drugs routinely used in medication, their cytotoxicity and anti-proliferation features are already widely documented and are consistent with those obtained in our work (Fig. 1). Moreover, the properties concerning the impairment of GAG synthesis via all three selected for this work compounds were verified, when used alone and in combination with genistein (Fig. 2). Interestingly, most significant inhibition of glycosaminoglycan production was observed in cells treated with a mix of nimesulide and genistein, resulted in an additive effect of these two compounds. The question remains what is the molecular mechanism by which the tested compounds reduce efficiency of GAG synthesis. It is known from the literature that analgesic and nonsteroidal anti-inflammatory drugs inhibit synthesis of prostaglandins^28^, and that prostaglandins function in cells as activators of intracellular signaling pathway through EGF receptor^29^. On the other hand, results of our previous studies suggested that genistein impairs GAG synthesis due to its inhibitory effect on the kinase activity of the epidermal growth factor receptor (EGFR)^7^. At this time, we showed that indomethacin and nimesulide can inhibit EGFR and PI3K signaling cascades (Figs 3 and 4). It appears that agents that target the EGFR/PI3K axes and signaling cascades show a considerable promise for treatment of MPS patients.

Our recent findings provided information on genistein targetome responsible for modulation of expression of GAG metabolism and lysosomal biogenesis and function genes^10^,^11^. In the work reported here, in turn, we observed that indomethacin and nimesulide can downregulate glycosaminoglycan production by blocking phosphorylation of EGF receptor (Figs 2 and 3), thus possibly impairing a signal transduction pathway necessary for activation of genes coding for enzymes involved in this anabolic process. The reduction in EGFR activity by tested NSAIDs, even more effective than by genistein (Fig. 3), may also be associated with the modulation of expression of genes which products act as activators of various intracellular signaling pathways. It was therefore interesting to study the effect of selected NSAIDs on cell transcriptome, with particular attention paid to GAG and lysosome-related genes’ activities, as well as to genes involved in synthesis of prostaglandins and various intracellular signaling pathways. We noticed some changes in expression of particular GAG metabolism-related and lysosomal function genes, with significant variation in the activity of several transcripts linked to intracellular signal transduction pathways and metabolism of DNA and proteins (Fig. 5, Tables 1 and 2). However, it should be noted at this point that essentially a rather subtle effect of the tested substances on the global activity of genome of human dermal fibroblasts was detected. These observations seem to be valuable particularly in the context of the safety in use of these compounds for humans.

The deregulation of GAGs may concern either all types of GAGs or some of them, depending on the agent being used. Our studies showed that the tested compounds, i.e. indomethacin, nimesulide and genistein exhibit deregulation of all types of glycosaminoglycans due to EGF receptor signaling cascades’ inhibition. In addition, in the case of cell treatment with NSAIDs, substances responsible for depletion of cellular sulphates, only sulphated GAGs (i.e. heparan sulfate, dermatan sulfate, chondroitin sulfate and keratan sulfate) seemed to be deregulated. Sulphates are basic and indispensable building blocks of GAGs. With the exception of hyaluronate, all glycosaminoglycans contain sulphate groups in ester linkages with the hydroxyl groups of the amino sugar residues. Nevertheless, in future study detailed quantification of every GAG individually will be of interest.

Summing up, the results presented in this work suggest that some of the non-steroidal anti-inflammatory drugs, and their combinations with isoflavone genistein, can be considered as a method for improvement of efficiency of therapy for mucopolysaccharidoses, especially their neurological forms. It is believed that our investigations can provide new information regarding the molecular mechanism of action of substances decreasing the synthesis of compounds being pathologically accumulated in the cells of people suffering from various types of genetically determined lysosomal storage diseases. On the other hand, proper choice of conditions of the drug applications are not trivial, and extensive studies are necessary for optimization of each particular combined treatment. Previous studies documented preliminary experiments with genistein-based SRT called GET-IT combined with ERT on MPS I culture cells as an effective approach^30^. Currently, the results of our studies on this kind of approach on MPS mouse models, conducted within the framework of another large research grant, are very promising. The same can be presumed on the development of a high efficacy of NSAID and ERT or HSCT combined therapies for MPSs, representing a promising method in indications of unmet medical needs. Understanding of these issues may result in serious progress in therapeutic approaches.

## Additional Information

**How to cite this article:** Mozolewski, P. *et al*. Nonsteroidal anti-inflammatory drugs modulate cellular glycosaminoglycan synthesis by affecting EGFR and PI3K signaling pathways. *Sci. Rep.* **7**, 43154; doi: 10.1038/srep43154 (2017).

**Publisher's note:** Springer Nature remains neutral with regard to jurisdictional claims in published maps and institutional affiliations.

## Figures and Tables

**Table 1:** Expression patterns of GAG metabolism- and lysosome-associated genes in 25 μM nimesulide, 100 μM genistein and mixture of them, for 24 h treated HDFa fibroblasts analyzed with microarray and real-time qRT-PCR custom panel.

Enzyme name	Enzyme function	Gene name	Microarray		Real-time qRT-PCR	
			FC ± SD			
			Nimesulide	Genistein*	Genistein	Nimesulide + Genistein
Acid phosphatase 5	conversion of orthophosphoric monoester to alcohol and orthophosphate	*ACP5*	1.0 ± 0.3	1.6 ± 0.1	1.3 ± 0.1	1.7 ± 0.4
Aspartylglucosaminidase	catabolism of N-linked oligosaccharides of glycoproteins	*AGA*	1.2 ± 0.1	1.9 ± 0.5	1.2 ± 0.1	1.5 ± 0.1
Arylsulfatase G	hydrolyze sulfate esters from sulfated steroids, carbohydrates, proteoglycans, and glycolipids	*ARSG*	n.d.	1.2 ± 0.4	1.9 ± 0.1	1.6 ± 0.2
Acid ceramidase 1	degradation of ceramide into sphingosine and free fatty acid	*ASAH1*	1.3 ± 0.2	1.7 ± 0.8	1.4 ± 0.2	1.6 ± 0.2
Chondroitin Sulfate Synthase 3	GAG synthesis, chain polymerization	*CHSY3*	n.d.	0.9 ± 0.2	0.7 ± 0.1	1.7 ± 0.2
Cathepsin K	bone remodeling and resorption	*CTSK*	n.d.	1.2 ± 0.2	1.2 ± 0.1	1.8 ± 0.1
Exostosin Glycosyltransferase 1	GAG synthesis, chain polymerization	*EXT1*	n.d.	0.6 ± 0.2	0.5 ± 0.0	0.5 ± 0.0
Alpha glucosidase	degradation of glycogen to glucose	*GAA*	n.d.	1.6 ± 0.7	1.3 ± 0.3	1.4 ± 0.4
Hexosaminidase A	degradation of the ganglioside GM2	*HEXA*	n.d.	1.4 ± 0.2	1.0 ± 0.1	1.3 ± 0.3
Heparan Sulfate Sulfotransferase 3A1	GAG synthesis, chain modyfication	*HS3ST3A1*	n.d.	0.5 ± 0.2	0.7 ± 0.1	0.6 ± 0.1
Hyaluronoglucosaminidase 3	degradation of hyaluronan	*HYAL3*	n.d.	1.7 ± 0.5	0.6 ± 0.0	0.6 ± 0.1
Beta mannosidase	N-linked glycoprotein oligosaccharide catabolism	*MANBA*	1.0 ± 0.3	2.4 ± 0.8	1.7 ± 0.2	2.2 ± 0.3
Sialyltransferase 4B	GAG synthesis, chain polymerization	*ST3GAL2*	1.1 ± 0.2	0.6 ± 0.1	0.5 ± 0.1	0.5 ± 0.1
Xylosyltransferase I	GAG synthesis, chain initiation	*XYLT1*	1.1 ± 0.1	0.6 ± 0.2	0.3 ± 0.1	0.4 ± 0.1

Values represent fold change (0.7 ≥ FC ≥ 1.3 for at least one of the conditions tested, n ≥ 3, with the *p*-value < 0.05), and denote differences for samples treated with tested compound or their mixture, against untreated samples, with respect to reference mRNA expression at a constant level.

n.d. stands for no data available, as no transcriptomic data regarding modulation of the expression of these genes were available due to the problem with low level analysis being related to elimination of the results for these transcripts after background adjustment.

^*^Stands for result based on raw data from Moskot *et al*.^11^.

**Table 2:** Table of selective gene sets enriched among genes significantly up-regulated (FC ≥ 1.3) by 10 μM indomethacin or 25 μM nimesulide after 24 h treatment of HDFa cells based on GSEA of Reactome pathway expression (NES, normalized enrichment score; FDR *q*-val < 0.25, *q* value of false discovery rate, and *p*-val < 0.01), with the signal transduction cascades and DNA and protein metabolism gene sets at the top.

Reactome overrepresented pathway	No. of genes	NES	FDR *q*-val
**10** **μM Indomethacin**			
SYNTHESIS OF DNA	84	1,868	0,254
DNA STRAND ELONGATION	29	1,851	0,148
S PHASE	101	1,848	0,113
SIGNALING BY WNT	64	1,597	0,249
DOWNSTREAM TCR SIGNALING	35	1,590	0,244
**25** **μM Nimesulide**			
ACTIVATION OF THE MRNA UPON BINDING OF THE CAP BINDING COMPLEX AND EIFS AND SUBSEQUENT BINDING TO 43S	60	1,817	0,208
FORMATION OF THE TERNARY COMPLEX AND SUBSEQUENTLY THE 43S COMPLEX	50	1,768	0,199
SYNTHESIS AND INTERCORVERSION OF NUCLEOTIDE DI AND TRIPHOSPHATES	19	1,721	0,237
TRANSLATION	155	1,711	0,199
YAP1 AND WWTR1 TAZ STIMULATED GENE EXPRESSION	24	1,707	0,168
SRP DEPENDENT COTRANSLATIONAL PROTEIN TARGETING TO MEMBRANE	115	1,706	0,147
UTR MEDIATED TRANSLATIONAL REGULATION	112	1,693	0,138
METABOLISM OF PROTEINS	441	1,666	0,158
REGULATION OF KIT SIGNALING	17	1,654	0,159
ANTIGEN PRESENTATION FOLDING ASSEMBLY AND PEPTIDE LOADING OF CLASS I MHC	20	1,653	0,148
INFLUENZA LIFE CYCLE	140	1,636	0,161
SYNTHESIS OF BILE ACIDS AND BILE SALTS VIA 7ALPHA HYDROXYCHOLESTEROL	15	1,599	0,196
CITRIC ACID CYCLE TCA CYCLE	23	1,597	0,190
OXYGEN DEPENDENT PROLINE HYDROXYLATION OF HYPOXIA INDUCIBLE FACTOR ALPHA	17	1,589	0,193
PEPTIDE CHAIN ELONGATION	90	1,585	0,184
TRANSPORT TO THE GOLGI AND SUBSEQUENT MODIFICATION	33	1,577	0,180
NONSENSE MEDIATED DECAY ENHANCED BY THE EXON JUNCTION COMPLEX	111	1,575	0,176
EARLY PHASE OF HIV LIFE CYCLE	18	1,554	0,207
INFLUENZA VIRAL RNA TRANSCRIPTION AND REPLICATION	106	1,554	0,199
